# Combined pharmacological administration of AQP1 ion channel blocker AqB011 and water channel blocker Bacopaside II amplifies inhibition of colon cancer cell migration

**DOI:** 10.1038/s41598-019-49045-9

**Published:** 2019-09-02

**Authors:** Michael L. De Ieso, Jinxin V. Pei, Saeed Nourmohammadi, Eric Smith, Pak Hin Chow, Mohamad Kourghi, Jennifer E. Hardingham, Andrea J. Yool

**Affiliations:** 1 Adelaide Medical School, University of Adelaide, Adelaide, SA 5005 Australia; 2 Oncology Department, The Basil Hetzel Institute, The Queen Elizabeth Hospital, Woodville, SA 5011 Australia

**Keywords:** Cell invasion, Collective cell migration, Target identification, Drug development

## Abstract

Aquaporin-1 (AQP1) has been proposed as a dual water and cation channel that when upregulated in cancers enhances cell migration rates; however, the mechanism remains unknown. Previous work identified AqB011 as an inhibitor of the gated human AQP1 cation conductance, and bacopaside II as a blocker of AQP1 water pores. In two colorectal adenocarcinoma cell lines, high levels of AQP1 transcript were confirmed in HT29, and low levels in SW480 cells, by quantitative PCR (polymerase chain reaction). Comparable differences in membrane AQP1 protein levels were demonstrated by immunofluorescence imaging. Migration rates were quantified using circular wound closure assays and live-cell tracking. AqB011 and bacopaside II, applied in combination, produced greater inhibitory effects on cell migration than did either agent alone. The high efficacy of AqB011 alone and in combination with bacopaside II in slowing HT29 cell motility correlated with abundant membrane localization of AQP1 protein. In SW480, neither agent alone was effective in blocking cell motility; however, combined application did cause inhibition of motility, consistent with low levels of membrane AQP1 expression. Bacopaside alone or combined with AqB011 also significantly impaired lamellipodial formation in both cell lines. Knockdown of AQP1 with siRNA (confirmed by quantitative PCR) reduced the effectiveness of the combined inhibitors, confirming AQP1 as a target of action. Invasiveness measured using transwell filters layered with extracellular matrix in both cell lines was inhibited by AqB011, with a greater potency in HT29 than SW480. A side effect of bacopaside II at high doses was a potentiation of invasiveness, that was reversed by AqB011. Results here are the first to demonstrate that combined block of the AQP1 ion channel and water pores is more potent in impairing motility across diverse classes of colon cancer cells than single agents alone.

## Introduction

Cancer is a leading cause of death worldwide, and accounted for 8.2 million deaths in 2012 according to the World Health Organization^1^, primarily due to metastasis^2,3^. Colorectal cancer is the second most common cancer in women and third in men^1^. A link between disease severity and upregulation of aquaporin-1 (AQP1) expression in certain cancer types has been documented previously^4,5^. AQP1 normally serves essential roles in fluid absorption and secretion in tissues including kidney, brain, eye and vascular system^6^. In aggressive cancers including subtypes of colorectal cancers, gliomas, lung adenocarcinoma, laryngeal cancer and cholangiocarcinomas, AQP1 channels are upregulated^7–11^. Levels of AQP1 expression show a positive correlation with angiogenesis, tumour progression, growth, migration and metastasis^12^. Knockdown of AQP1 with small-interfering RNAs substantially impaired cancer cell migration *in vitro*^13^. Conversely, transfection of AQP1 into deficient lines (B16F10 melanoma, and 4T1 mammary gland tumour) accelerated cell migration *in vitro* and increased the likelihood of lung metastases in mice *in vivo*^14^. Colon cancer cells (HT20) transfected with AQP1 similarly exhibited increased cell migration rates and enhanced extravasation after injection via the tail vein in mice^15^. AQP1 channels have been proposed to facilitate extension of the leading edges (lamellipodia) of migrating cells to speed the rate of movement^16^.

Inhibition of AQP1 channel activity is a strategy of interest for potential therapeutic control of metastasis in AQP1-expressing cancers. In AQP1-dependent cancer lines, other mammalian water channels such as AQP4 do not substitute for AQP1 in facilitating movement^17^, suggesting the migration-enhancing property relies on more than water permeability. Converging lines of evidence support the idea that AQP1 is dual water and ion channel, mediating osmotic water flow through individual subunit pores, and cation conductance through the central pore of the tetramer^18–20^. The non-selective monovalent cation conductance is gated by cGMP and depends on loop D structural integrity for channel activation^19,21–24^. In some expression systems, AQP1 ion channels have a low opening probability^25^ or are not detectable^26^, suggesting gating of AQP1 is subject to additional regulation such as tyrosine phosphorylation^27^. The idea that the AQP1 cation pore at the four-fold axis of symmetry is separate from the individual water pores in each monomer^19,27,28^ is supported by differences in pharmacological sensitivities and differential effects of site-directed mutations^25,29,30^. There is a gap in knowledge regarding the properties of AQP1 that permit its migration-enhancing effect, but the inability of other water-selective channels to substitute suggests the dual ion and water channel activities of AQP1 might be involved^31^. The discovery of pharmacological modulators of AQP1 has allowed dissection of the mechanisms of action of AQP1 in cell migration at a level not possible previously.

Pharmacological modulators of the AQP1 ion conductance include the agonist cyclic GMP^21^, and antagonists cadmium^32^, calcium^33^ and pharmacological derivatives of bumetanide such as AqB007 and AqB011^34^. Blocking native AQP1 ion channels in choroid plexus with Cd^2+^ was shown to alter net cerebrospinal fluid production *in vitro*^32^. AqB011 is a bumetanide derivative that selectively blocks the ion pore of AQP1, binding at the intracellular loop D gating domain^24^ without affecting water fluxes, and slows the migration of AQP1-expressing cancer cells^34^. Pharmacological modulators of the AQP1 water pore include the antagonists mercury^35^, tetraethylammonium^29,36,37^, metals^38^, AqB013^39^ and bacopasides^40^, and the agonist AqF026^30^. The inhibitor AqB013 and a related compound, AqB050, have been shown to reduce cancer cell migration rates *in vitro*^41,42^. The natural medicinal plant product, Bacopaside II, isolated from *Bacopa monnieri* appears to dock in the cytoplasmic vestibule of the AQP1 water pore, occluding water flux without affecting the AQP1 ion conductance, and slows cell migration in an AQP1-expressing colon cancer line^40^.

Prior reports have focused on measuring effects of single AQP1 modulators using two-dimensional wound closure assays of cancer lines. This study is the first to assess synergistic actions of AQP1 ion and water channel inhibitors applied together, and to evaluate effects on three-dimensional invasion through extracellular matrix. The two human colorectal adenocarcinomas cell lines with epithelial morphologies selected for comparison were: HT29 with high levels of AQP1 expression, and SW480 with low levels of AQP1 expression^40,43^. Results here showed that combined administration of AQP1 water and ion channel blockers produced an amplified block of colon cancer cell migration in both colon cancer lines. Inhibition of the AQP1 ion channel reduced cancer cell invasiveness. The relative efficacy of the AQP1 inhibitors was dependent on the abundance and localization of AQP1 protein in the plasma membranes, which was greater in HT29 than in SW480 cells. In summary, AQP1 water and ion fluxes appear to have a coordinated role in facilitating AQP1-dependent cancer cell migration. Simultaneous targeting of both the water and ion channel functions of AQP1 appears to offer opportunities to control cancer metastasis at lower doses and across more diverse classes of cancers than would be possible with single agents alone.

## Results

### AQP1 expression and localization in HT29 and SW480 cell lines

**Figure 1:**
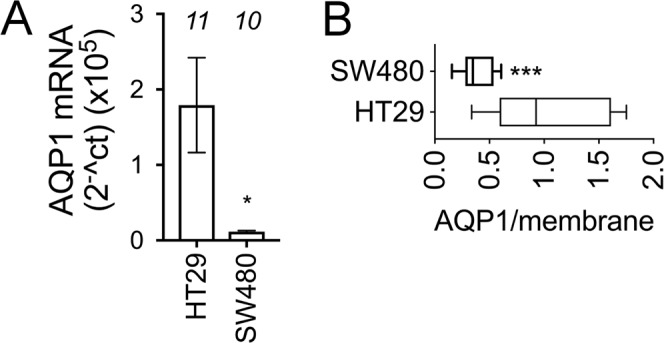
AQP1 transcript and membrane expression levels were higher in HT29 cells than SW480 cells. (**A**) AQP1 mRNA levels in HT29 cells (n = 11) and SW480 cells (n = 10), as determined by qRT-PCR. (**B**) Ratios of signal intensity (anti-AQP1 to membrane dye), in HT29 and SW480 cells showing relative levels of membrane AQP1 expression. See methods for statistical analysis details.

Levels of AQP1 expression were quantified previously in HT29 and SW480 cell lines by western blot and quantitative real-time reverse-transcription polymerase chain reaction (qRT-PCR), and showed that AQP1 transcript and protein levels were significantly higher in HT29 than in SW480 cells^40,43^. Quantitative PCR on the same passages of cells used in the present study demonstrated a fifteen-fold higher level of AQP1 transcript in HT29 as compared to SW480 cells (Fig. 1A), confirming prior results. Confocal imaging demonstrated that HT29 further exceeded SW480 in AQP1 levels when the subcellular distribution in the plasma membrane was considered. Membrane-associated AQP1 protein was almost three-fold higher in HT29 cells than in SW480 cells (Fig. 1B). Amplitudes of colocalized plasma membrane and AQP1 fluorescence signals were significantly lower in SW480 (0.38 ± 0.04; n = 6) than in HT29 (1.05 ± 0.15) cells.

**Figure 2:**
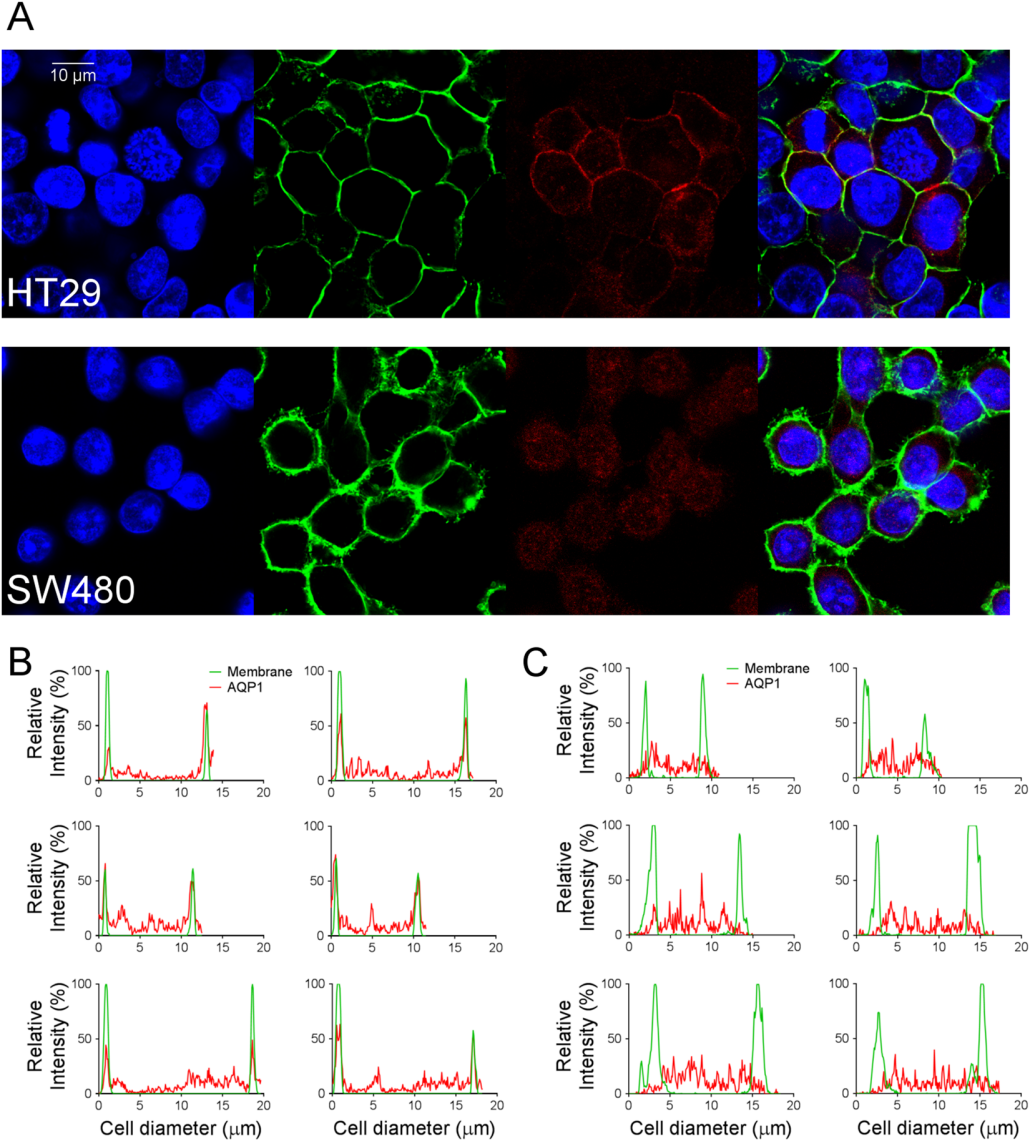
Confocal images and quantitative analyses of AQP1 subcellular localization measured by immunolabelling. (**A**) Confocal images of a single field of view for HT29 (top row) and SW480 (bottom row) cells. The panels in each row (from left to right) depict nuclear staining (blue); membrane staining (green); AQP1 signal (red); and an overlay of the three images (right). (**B,C**) Each graph shows the intensities (y-axis) of the membrane stain (green) and the AQP1 signal (red), as a function of distance across the diameters of individual HT29 (**B**) or SW480 (**C**) cells, for two cross-sections per cell.

AQP1 signal localization in HT29 and SW480 cells was assessed in greater detail by immunofluorescent labelling of AQP1 in combination with a fluorogenic membrane dye (MemBrite™), and Hoechst nuclear stain (Fig. 2A). Using Fiji software (ImageJ, National Institutes of Health), intensities were quantified for anti-AQP1 and membrane dye signals, and plotted as a function of cross-sectional distance for six transects in each cell line (Fig. 2B,C). Anti-AQP1 signals showed a robust correlation with the membrane signal in HT29 cells, whereas in SW480 cells the AQP1 signals were predominantly in the submembrane and cytoplasmic domains.

### Combined treatment with bacopaside II and AqB011 produced enhanced block of wound closure in colon cancer cells

**Figure 3:**
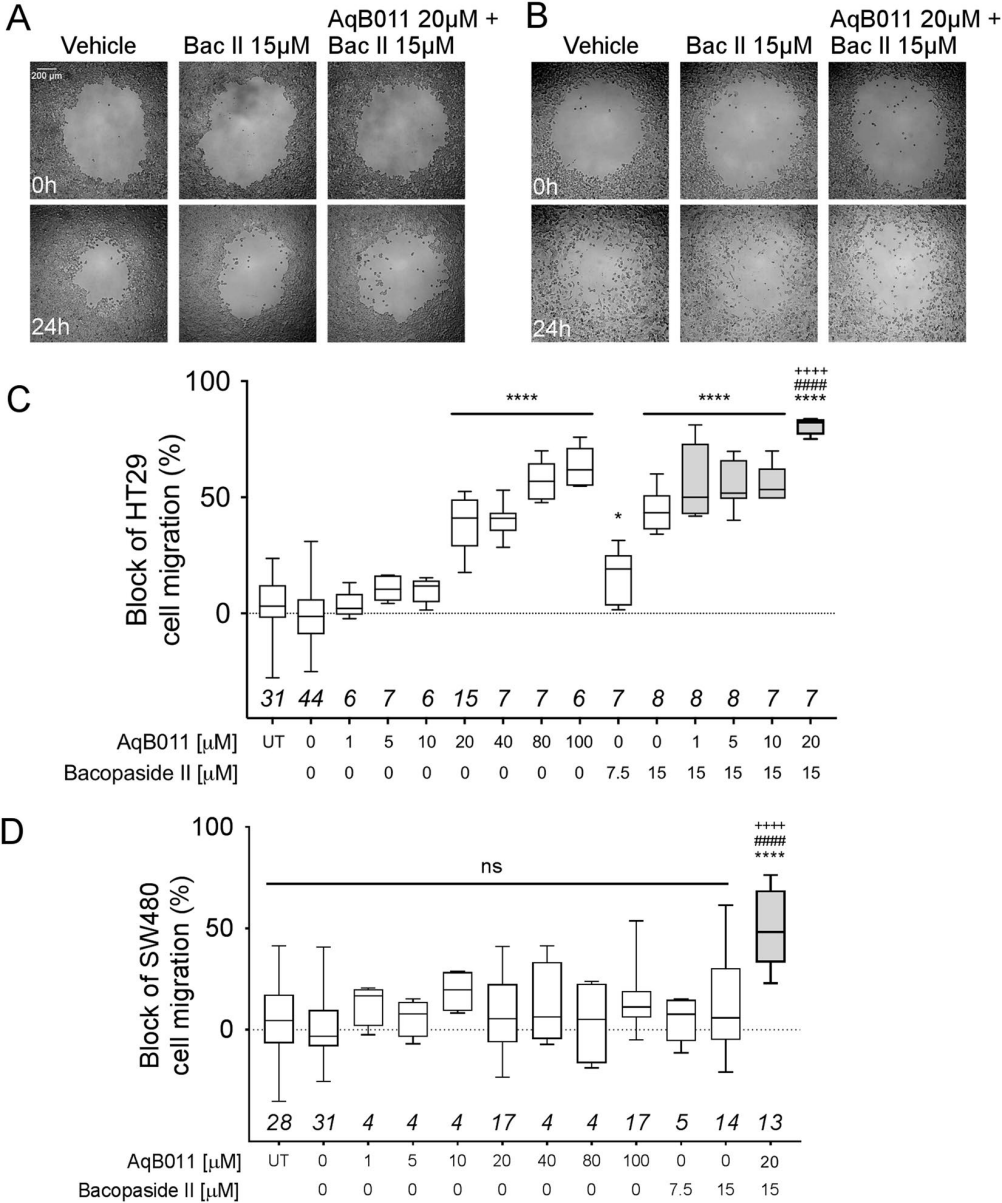
Wound closure assay showing the block of cell migration in HT29 cells by bacopaside II, AqB011 and combined treatments. (**A**) Representative images showing HT29 cells treated with vehicle, bacopaside II (15 μM), and combined treatment at 0 hours (upper row) and 24 hours (bottom row). Scale bar applies for both (**A,B**). (**B**) Representative images showing SW480 cells treated with vehicle, bacopaside II (15 μM), and combined treatment at 0 hours (upper row) and 24 hours (bottom row). (**C**) Box plot depicting percent block (%) of HT29 wound closure after 24 hours. White bars represent single treatments; grey bars represent combined treatment with AqB011 and bacopaside II. AqB011 (20 μM) and bacopaside II (15 μM) treatment yielded block that was significantly greater than vehicle (****), AqB011 (20 μM) alone (####), and bacopaside II (15 μM) alone (++++), suggesting an additive interaction; n-values are in italics above the x-axis for (**C,D**). (**D**) Box plot depicting wound closure block (%) for SW480 after 24 hours. White bars represent single treatments; grey bars represent combined treatment with AqB011 and bacopaside II. SW480 cells were insensitive to block by AqB011 or bacopaside II alone. AqB011 (20 μM) and bacopaside II (15 μM) combined treatment yielded block that was significantly greater than vehicle (****), AqB011 (20 μM) alone (####), or bacopaside II (15 μM) alone (++++).

The effects of AqB011 and bacopaside II alone and combined on two-dimensional migration of HT29 and SW480 cells were tested using circular wound closure assays (Fig. 3A,B). In HT29 cells at 24 h, significant impairment of migration was observed with AqB011 alone (at 20 to 100 µM), or bacopaside II alone (at 7.5 and 15 µM), as compared to vehicle control (Fig. 3C), confirming previous findings^34,40^. With agents applied singly, migration was reduced 38% by AqB011 (20 µM), and 44% by bacopaside II (15 µM). However, the combined treatment produced a block of 81%, significantly greater than with either agent alone. Conversely, SW480 cells showed no appreciable sensitivity of migration to individual AqB011 or bacopaside II treatments (Fig. 3D), again confirming previous findings^34,40^. However, the combined treatment of SW480 cells with AqB011 (20 µM) and bacopaside II (15 µM) resulted in a 50% block of migration, showing that the combination of inhibitors was more potent than either agent alone. These data indicate AqB011 and bacopaside II applied in combination can slow migration in a line that would otherwise escape control by single agents alone at non-cytotoxic doses.

Timelapse video files show the migration processes in vehicle control and treatment groups in more detail (Supplementary Videos ‘HT29’ and ‘SW480’).

### Cell viability was not affected by AqB011 and bacopaside II

**Figure 4:**
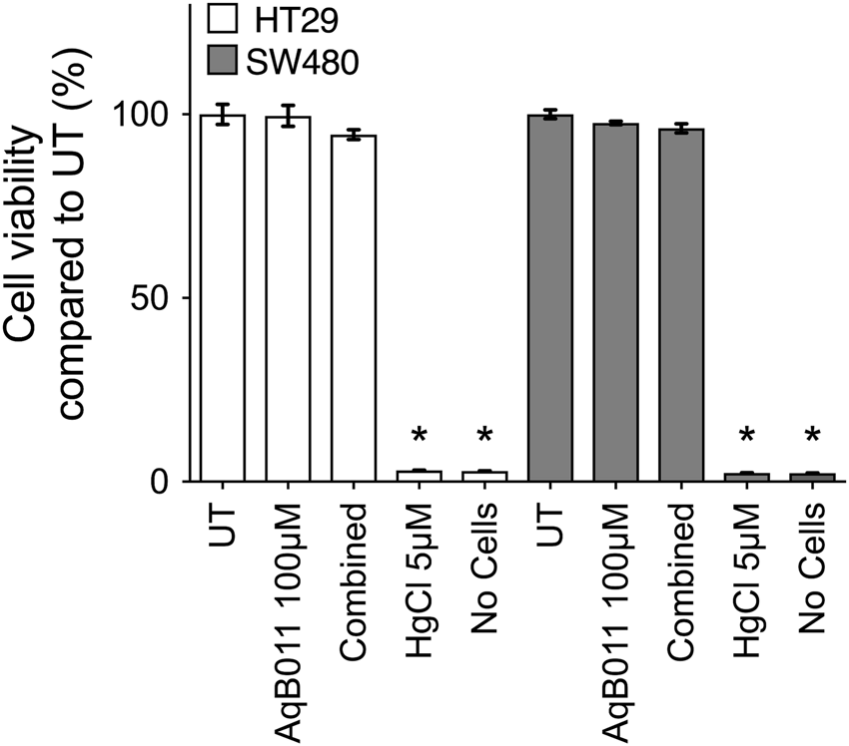
Summary histogram of the effects of pharmacological treatments on viability measured by alamarBlue assay for HT29 and SW480 cells. ‘Combined’ indicated cotreatment with AqB011 (20 µM) and bacopaside II (15 µM). Mercuric chloride served as a positive control for cell death; ‘no-cell’ controls confirmed minimal background fluorescence. All treatments were *n* = 4. Data were standardized to results for untreated (UT) cells.

Cell viability data measured with the alamarBlue assay were standardized to results for untreated groups in each cell line (Fig. 4). HT29 cell viability was 99.6 ± 1.4% in AqB011 (100 µM), and 94.5 ± 0.7% in combined treatment. In contrast, cytotoxic mercuric chloride (5 µM) resulted in 3.1 ± 0.04% viability. SW480 cell viability was 97.6 ± 0.2% in AqB011 (100 µM), 96.2 ± 0.6% in combined treatment, and 2.4 ± 0.04% in mercuric chloride (5 µM). The lack of effects of bacopaside II and AqB011 on cell viability demonstrated that cytotoxicity did not account indirectly for the reduced cell migration.

### Knockdown of AQP1 reduced the sensitivity of HT29 cells to block by bacopaside II and AqB011

**Figure 5:**
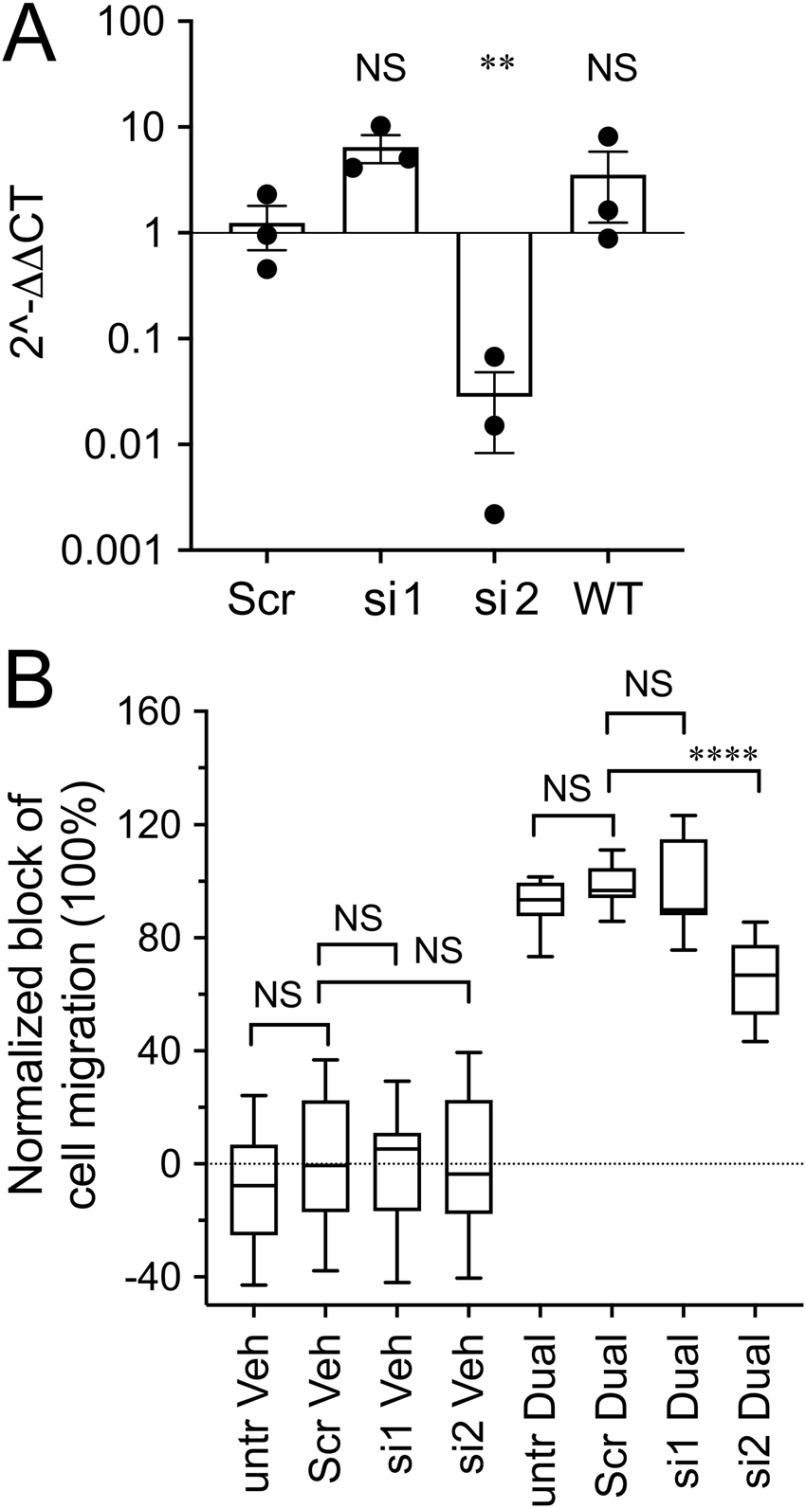
Effects of siRNA knockdown of AQP1 on the pharmacological sensitivity of HT29 cell migration to block by the combined inhibitors AqB011 and bacopaside II. (**A**) AQP1 siRNA2 (si2) but not siRNA1 (si1) significantly decreased AQP1 mRNA levels, as compared with scrambled control siRNA (Scr) and untreated (WT) in HT29 cells (n = 3 per group). (**B**) Box plot showing the decreased sensitivity of HT29 cell migration to pharmacological block after siRNA knockdown of AQP1. Values for HT29 wound closure at 24 h were standardized as a percentage of the scrambled control treated with combined inhibitors (Scr Dual; 100%), specifically to illustrate the differences in pharmacological effectiveness of the combined agents in siRNA1, siRNA2, and untreated. The effectiveness of the combined inhibitors was significantly impaired only in the siRNA2-transfected group (si2 Dual). siRNA1 (si1 Dual), scrambled (Scr Dual) and non-transfected (untr Dual) groups showed no loss of sensitivity to the combined inhibitors (n = 14 per group).

The role of AQP1 as a primary target of action for the combined inhibitors was tested using siRNA transfection with two different AQP1-siRNA sequences (siRNA1 and siRNA2), as compared with scrambled controls. Figure 5A shows that siRNA2 significantly reduced AQP1 transcripts levels (~44 fold decrease), as compared to scrambled control siRNA. In contrast, siRNA1 was not effective in knocking down AQP1. Matched scrambled controls also had no effect on AQP1 transcript levels.

AQP1 knockdown via siRNA has been shown previously to reduce cell migration^44–46^. In Fig. 5B, in order to highlight differences in pharmacological sensitivity that were due specifically to siRNA effects, all results for percent block were standardized to the mean percent block measured in the scrambled siRNA control group (Scr Dual, set by definition as 100%). Only siRNA2 significantly impaired the effect of the combined inhibitors in slowing migration. The other groups (untreated, siRNA1) showed no change in pharmacological sensitivity as compared with scrambled siRNA. At 24 hours, vehicle-treated cells transfected with scrambled siRNA achieved 20 ± 1% wound closure. Vehicle-treated cells transfected with AQP1 siRNA1 achieved 17 ± 1% wound closure. When cells transfected with scrambled siRNA were treated with combined inhibitors, wound closure was almost completely blocked (0.5 ± 0.4%). The cells transfected with AQP1 siRNA2 retained a greater capacity for wound closure in the presence of the combined inhibitors (6 ± 0.6%), demonstrating a reduced pharmacological sensitivity to the AQP1 inhibitors. These results suggested that the effectiveness of the inhibitors on cell migration correlated with the level of AQP1 expression.

### Live cell imaging of bacopaside II and AqB011 effects on individual cell migration trajectories

**Figure 6:**
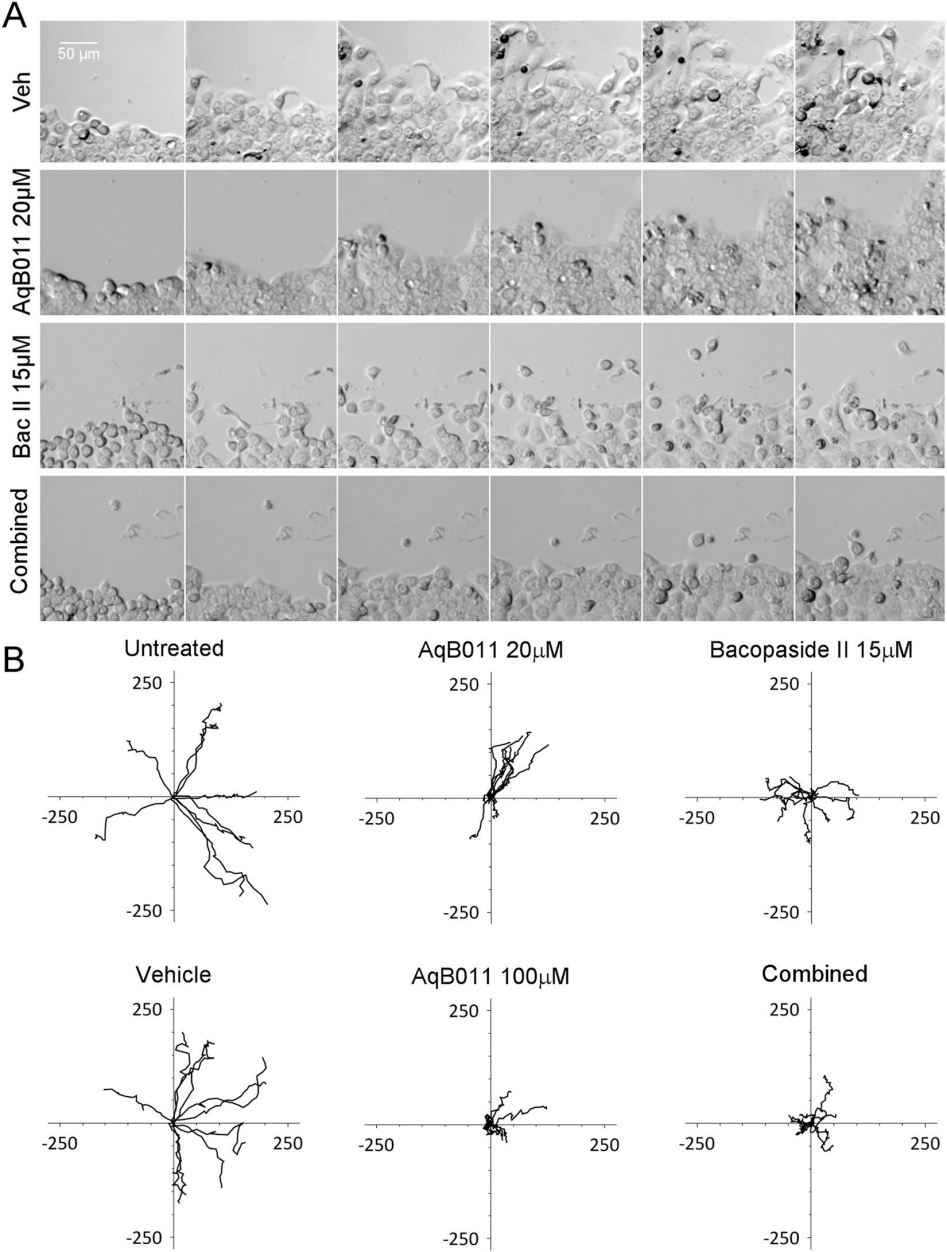
Live cell imaging of HT29 cells and plots of migration trajectories in treatments with bacopaside II and AqB011 alone, and in combination. Single cells at the boundaries of circular wounds were tracked with time-lapse images taken at 30 minute intervals for 24 hours at 37 °C. (**A**) Panels of six images each from time-lapse series are shown at 4-hour intervals, for treatments with vehicle, AqB011 (20 μM), bacopaside II (15 μM), and combined AqB011and bacopaside II. (**B**) Trajectory plots of individual cells (n = 9–11 per treatment group), monitored by the position of the cell nucleus at 60 minute intervals over 24 hours. Movement was referenced to the starting position set as 0,0 on graph axes; X and Y units are μm.

**Figure 7:**
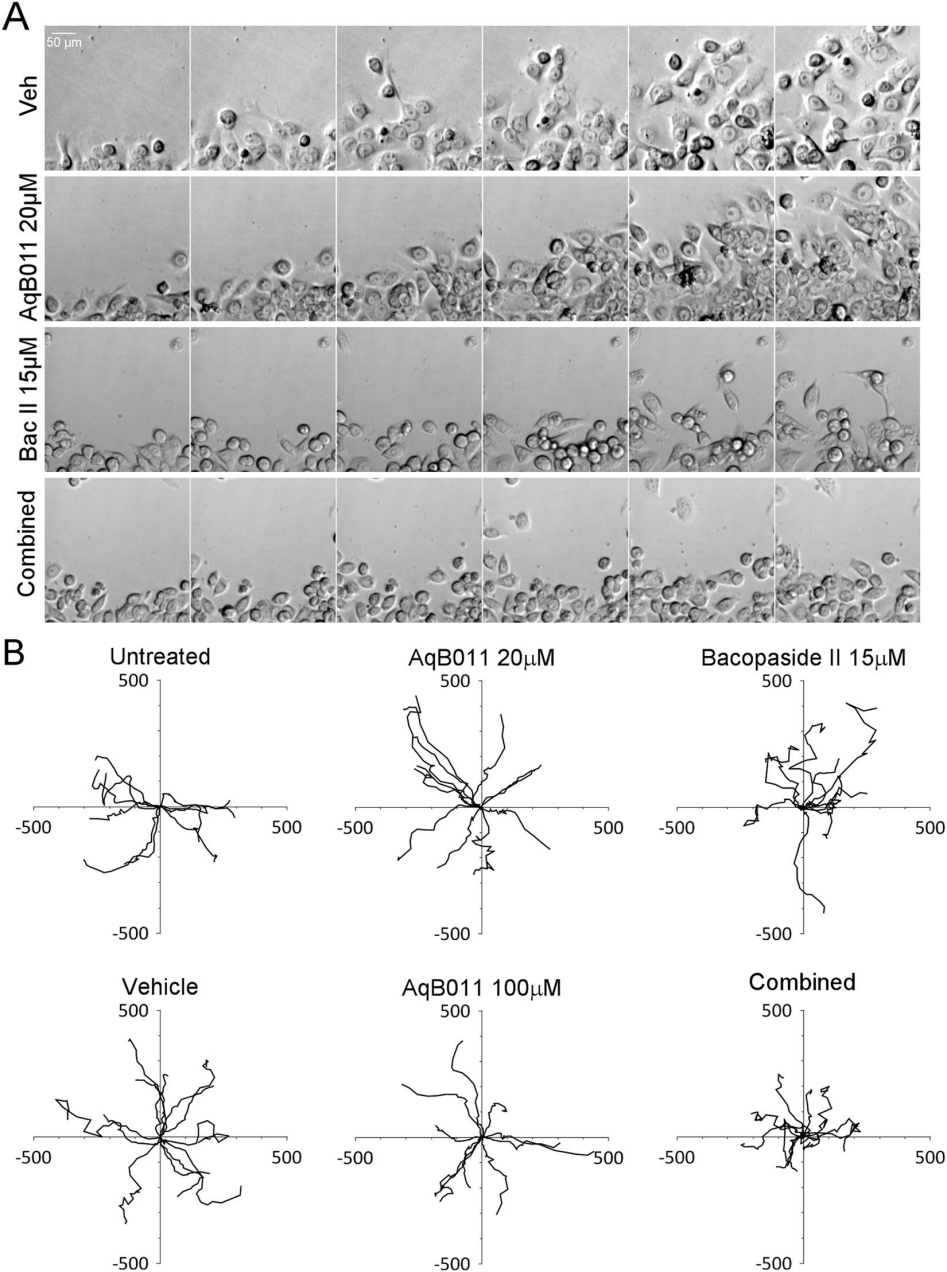
Live cell imaging of SW480 cells and plots of migration trajectories in treatments with bacopaside II and AqB011 alone, and in combination. Single cells at the boundaries of circular wounds were tracked with time-lapse images taken at 30-minute intervals for 24 hours at 37 °C. (**A**) Panels of six images each from time-lapse series are shown at 4-hour intervals, for vehicle, AqB011 (20 μM), bacopaside II (15 μM), and combined (Combined) treatment groups. (**B**) Trajectory plots of individual cells (n = 10–12) per treatment group, monitored by the position of the cell nucleus at 60 minute intervals over 24 hours; X and Y values are in μm.

The effects of the AQP1 modulators on individual cell trajectories were tracked over 24 hours for HT29 (Fig. 6), and SW480 (Fig. 7) cells using live cell imaging. In HT29 cells, slowed wound closure with AQP inhibitors was evident in time lapse images of the wound edge (Fig. 6A). Impaired velocity and direction of migration were evident in the short convoluted trajectories in the treatment groups as compared with untreated and vehicle controls (Fig. 6B). In contrast, SW480 cells showed normal wound edge closure (Fig. 7A) and migration trajectories (Fig. 7B) in the treatment groups with single agents; however the combined agents appeared to reduce the total distance travelled, yielding shorter trajectories.

**Figure 8:**
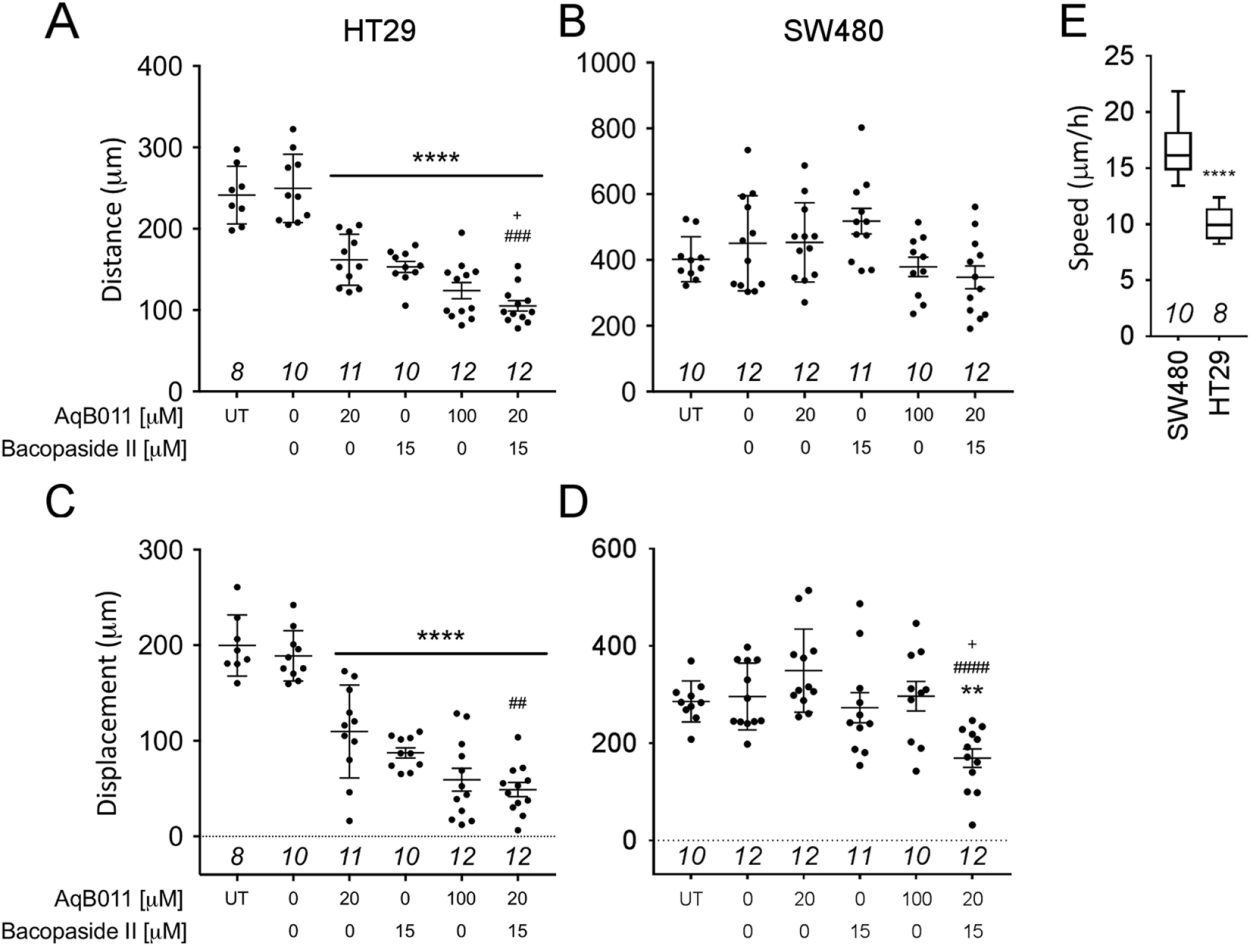
Summary plots of total distances travelled and net displacement of HT29 and SW480 cells in each treatment group, measured by live cell imaging. (**A**) 20 μM AqB011 or 15 μM bacopaside II treatments alone significantly reduced the total distance migrated by HT29 cells as compared to vehicle-treated (0 AqB011, 0 Bacopaside II). The level of inhibition was significantly enhanced in the combined treatment as compared with either agent alone (###). (**B**) No significant block of SW480 total distance was seen in any treatment as compared with vehicle. **(C**) Net displacement in HT29 cells was reduced by all treatments as compared to vehicle. Combined treatment showed a significantly greater block of HT29 net displacement than either treatment alone (##). (**D**) Only the combined treatment reduced SW480 net displacement as compared to vehicle, or as compared to either treatment alone (####). (**E**) SW480 mean migration velocity was significantly greater than that of HT29. Distance and displacement measures for untreated (UT) showed no significant differences from vehicle-treated in all panels (**A–D**; symbols not shown). *n*-values in italics are above the x-axis.

Total distance travelled (the cumulative length of the trajectory) and net displacement (the difference in position between the starting and ending point) per cell were quantified from analyses of live cell imaging results (Fig. 8). HT29 cells showed a dose-dependent effect of AqB011 in decreasing total distance travelled as compared with vehicle-treated cells (Fig. 8A). Bacopaside II at 15 µM also significantly reduced total distance. Combined AqB011 (20 µM) and bacopaside II (15 µM) was more effective than either agent alone in HT29 cells. In contrast, SW480 showed no effect of AqB011 alone (Fig. 8B); the total distance after treatment with 100 µM AqB011;was not significantly different from vehicle-treated cells. Similarly, bacopaside II (15 µM) alone had no appreciable effect on SW480 cell total distance travelled. Combined AqB011 (20 µM) and bacopaside II (15 µM) treatment appeared to reduce total distance travelled, but the difference was not statistically significant.

Net displacement over 24 h was significantly reduced in all treatment groups as compared with vehicle in HT29 cells (Fig. 8C). In SW480 cells, a significant decrease of net displacement (Fig. 8D) was seen only with the combined pharmacological treatment. Bacopaside II alone or AqB011 alone yielded net displacement values in SW480 cells comparable to vehicle-treated cells. The overall distances travelled were greater in SW480 than HT29 cells, illustrating an inherently higher two-dimensional migration velocity (Fig. 8E) with a low sensitivity to AQP1 modulators, indicating mechanisms other than AQP1 can enable motility in some cancer lines. However, the significant impairment of net displacement suggested that the directionality of movement (i.e., the ability to maintain a consistent vector over successive intervals) was compromised by the combined treatment in both cell lines.

### Effects of bacopaside II and AqB011 on lamellipodial formation

**Figure 9:**
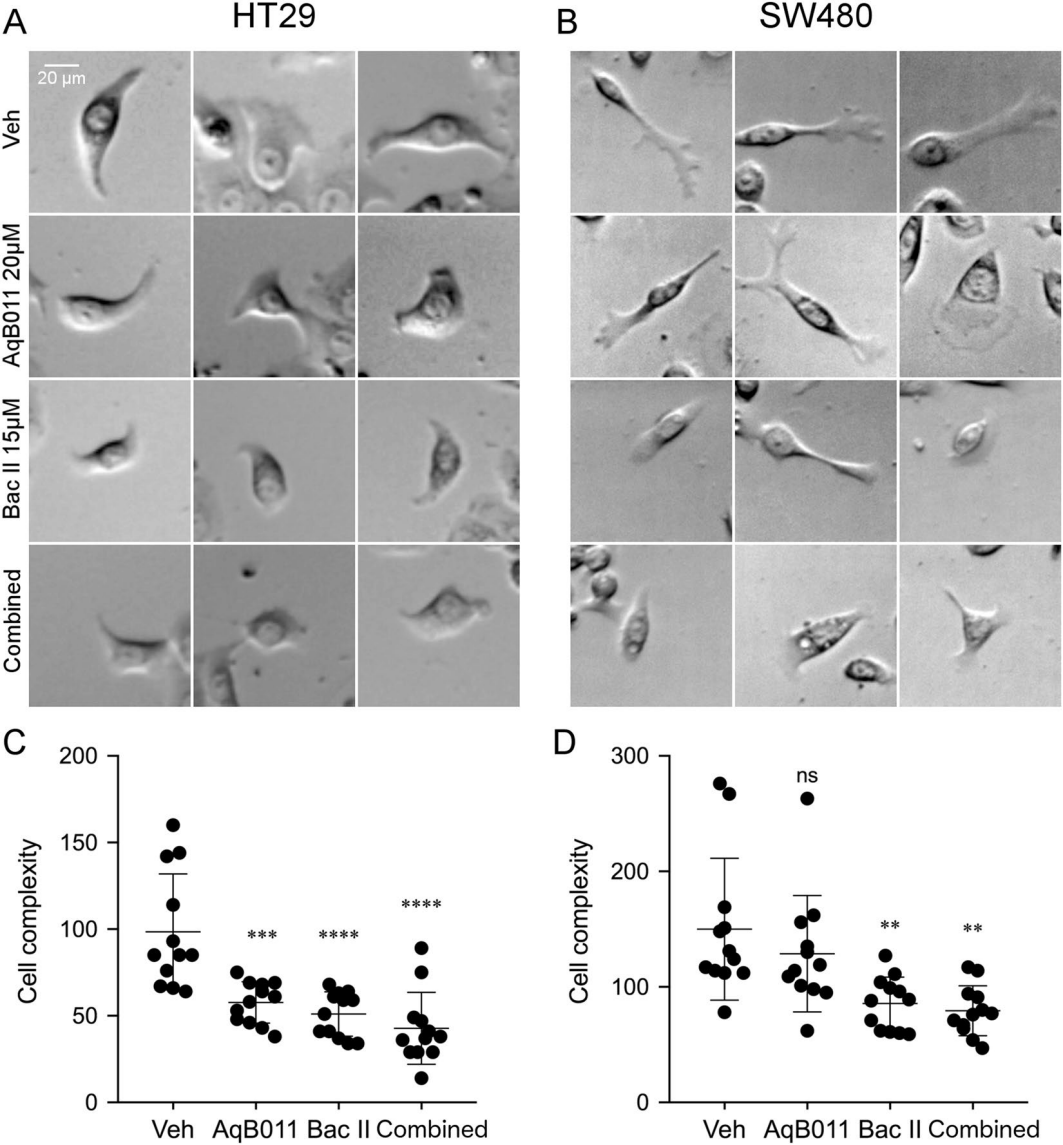
Images of actively migrating HT29 and SW480 cells in different treatment groups, showing three representative examples each. Vehicle-treated HT29 (**A**) and SW480 (B) cells (top row) showed distinct membrane protrusions. AqB011 and bacopaside II reduced protrusion sizes of HT29 cells as compared to vehicle in all treatment groups (**A**; rows 2–4). For SW480 cells, cellular protrusions appeared smaller in bacopaside II (B; rows 3 and 4) but not changed by AqB011 (**B**; row 2). “Cell complexity” values were quantified as the summed lengths of skeletonized morphological structures for indivual cells (see Methods for details) located at wound frontier positions for HT29 (**C**) and SW480 (**D**) cells (n = 12 per group). (**C**) HT29 cells treated with 10 μM AqB011 (AqB011), 15 μM bacopaside II (Bac II) or combined treatment (Combined) showed significant decrease in cell complexity compared with vehicle control (Veh). (**D**) In SW480 cells, bacopaside II and combined treatment decreased cell complexity as compared with vehicle control (Veh), but AqB011 had no appreciable effect.

Cell migration typically involves the formation of membrane protrusions such as actin-rich lamellipodia, which extend in the direction of locomotion and provide a base on which cells move forward^12,47^. A loss of lamellipodial structures has been associated with reduced rates of migration^48^. Images of representative cells illustrate the differential effects seen with single and combined inhibitor treatments in HT29 (Fig. 9A) and SW480 (Fig. 9B) cells. Vehicle-treated control groups in both cell were characterized by a high proportion of cells with membrane protrusions. Most HT29 control cells had flat sheet-like protrusions or bipolar wing-like processes. Most SW480 control cells had a primary lamellipodium that was slender, long, and ramified into a flattened lobed protrusion at the distal end.

In all treatments (bacopaside II and AqB011, alone and combined), HT29 lamellipodia were reduced in length as compared to vehicle-treated cells (Fig. 9A), and in frequency, seen as a smaller proportion of the cell population that showed processes. In contrast, SW480 lamellipodial extensions appeared to be unaffected by AqB011. However SW480 extensions were reduced in size and number in treatments with bacopaside II alone, or combined agents (Fig. 9B).

To quantify morphological data objectively, a score for ‘cell complexity’ was developed. Computer-based analyses were used to transform single cell images into skeletonized segments connected in branchworks to represent the morphological structures including cellular processes. Scores for cell complexity were calculated as the summed lengths of segments per cell (Fig. 9C,D). Simple circular cells were captured by a single line across the diameter, giving a low score. Complex cells with multiple or branched processes required a branchwork of skeletonized segments, giving a higher score. Scores in Arbitrary Units (AU) are based on the number of pixels. In HT29, cell complexity scores were significantly lower in all treatment groups, with AqB011 (mean 58 ± 3 AU), bacopaside II (51 ± 4), or combined (43 ± 6), as compared to vehicle control (98 ± 10). In SW480, cell complexity scores in AqB011 (129 ± 15) were not significantly different from vehicle control, but were reduced in bacopaside II (86 ± 7) and combined groups (79 ± 6), as compared to vehicle (150 ± 18). In summary, AqB011 reduced cell complexity scores in HT29 but not SW480 cells; bacopaside II reduced cell complexity scores for both cell lines; and combined treatment reduced complexity scores for both cell lines. The treatments which compromised lamellipodial structures were consistent with the loss of directional movement measured by net displacement (Fig. 8).

### AqB011, but not bacopaside II, inhibits colon cancer cell invasiveness

**Figure 10:**
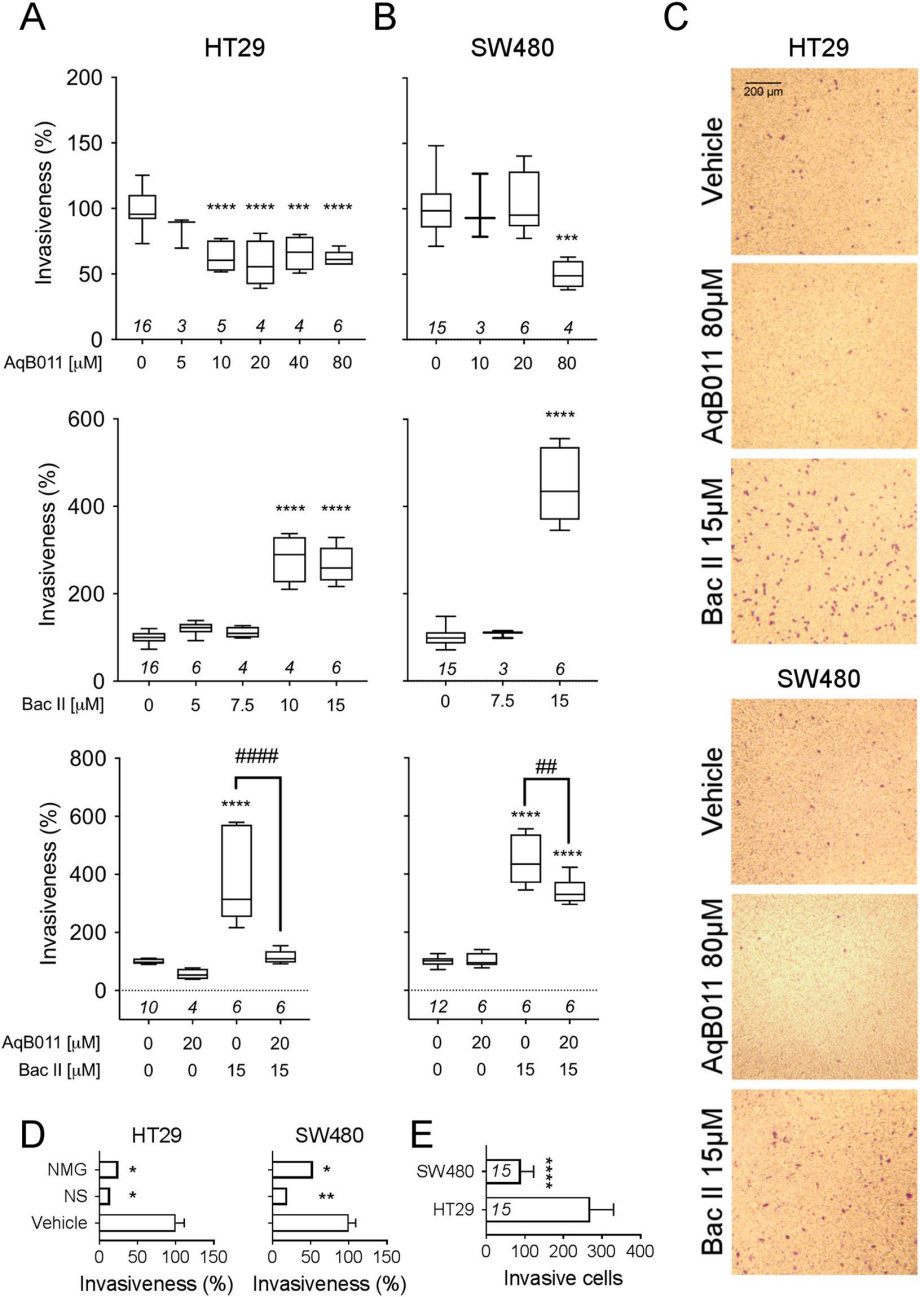
Transwell invasiveness of HT29 and SW480 cells in treatments with bacopaside II, AqB011, and combined agents. Box plots depicting cell invasiveness at 24 hours for HT29 (**A**) and SW480 (**B**) cells treated with AqB011 (row 1), bacopaside II (row 2), or both (row 3). (**A**) HT29 cell invasiveness was inhibited by AqB011 (10 to 80 μM). In contrast, bacopaside II (10 and 15 μM) potentiated HT29 invasiveness. Combined treatment blocked invasiveness, overriding the potentiation effect. (**B**) SW480 cell invasiveness was inhibited by AqB011 only at the highest dose tested (80 μM), and potentiated by bacopaside II (15 μM). Combined treatment partially counteracted the potentiation effect. (**C**) Images of HT29 and SW480 show cells that successfully crossed the ECM-like layer to reach the trans-side of the chamber in treatments with vehicle, AqB011 80 μM, or bacopaside II 15 μM. (**D**) Histograms showing the reduced invasiveness of HT29 and SW480 cells in the absence of ECM (no matrix gel; NMG), or in the absence of a serum gradient (no serum; NS), as compared with vehicle controls (vehicle) having both a serum gradient and ECM. (**E**) Untreated HT29 cells were significantly more invasive than SW480 cells. *n*-values are shown in italics above the y-axis (or in panel E, within the histogram bars).

In cancer metastasis, cells move in three dimensional space across tissue boundaries and through extracellular matrix (ECM), involving attributes that are not fully captured in two-dimensional wound closure models. The effects of AQP1 inhibitors were tested on transwell invasion through an ECM layer on a semi-permeable filter towards a chemoattractant (FBS), quantified by staining and counting migrated cells at set times (Fig. 10).

Treatment with AqB011 alone resulted in approximately 40% reduction of HT29 cell invasiveness at concentrations ≥10 µM (Fig. 10A; row 1). The highest dose of AqB011 (80 µM) also impaired SW480 cell invasiveness; lower doses had no significant effect (Fig. 10B; row 1). Unexpectedly, higher doses of bacopaside II alone potentiated invasiveness in both cell lines (row 2), increasing transwell movement by 2.7 fold in HT29 cells, and 4.5 fold in SW480 cells. Bacopaside II potentiation in HT29 cells was reversed in full by co-application of AqB011 (Fig. 10A, row 3), and partially reversed in SW480 (Fig. 10B, row 3). Transwell invasion required the presence of a chemotactic gradient (FBS), and an ECM gel layer (Fig. 10D). Even though the average surface migration velocity was slower for HT29 cells (Fig. 8E), their capacity for invasion (269 ± 16 cells per field of view; n = 15) was significantly greater than SW480 (90 ± 8 cells per field of view; n = 15) (Fig. 10E).

This work is the first to show the anti-invasive effects of AQP1 ion channel blocker AqB011 in colon cancer cells, suggesting a novel role for AQP1 ion conductance in facilitating cancer invasion. HT29 was more sensitive to the inhibitory effect of AqB011 than SW480, consistent with observations from 2D assays. In contrast, bacopaside II surprisingly enhanced cancer cell invasion in both cell lines, suggesting this agent is likely to have multiple targets of action, and that its side effects are mediated at least in part at a level upstream of AQP1-dependent signalling.

## Discussion

Cell migration and invasion are key pathological processes in cancer metastasis. Upregulation of AQP1 expression has been shown to enhance cell migration and metastasis in certain subtypes of cancers^13–15^. In other types of cancers, AQP3, AQP4, or AQP5 are increased in expression and influence viability, proliferation and migration^12,49–52^. Process formation during migration is thought to be driven by reversible assembly of actin filaments^53–55^, and local cell volume increases facilitated by water and ion fluxes^31,56,57^. To achieve a paired water and ion flux at the membrane leading edges, classes of AQPs that are upregulated in cancers would in theory be dual water and ion channels themselves, or water channels that can be co-localized with ion transporters or channels. AQP1-mediated water influx at leading edges, leading to local volume changes associated with process extension and cell movement^31^, enhances cancer cell motility and invasion. In glioma cells AQP4 colocalizes with the chloride channel (ClC2) and the potassium-chloride co-transporter 1 (KCC1), which could provide a driving force for water efflux leading to cell shrinkage, augmenting invasiveness^58,59^. AQP5 is not known to have an ion channel function^21^, but can colocalize with ion channels or transporters such as the Na^+^/H^+^ exchanger in breast cancer cells^56^.

Results here showed that efficacy of AqB011 in impairing motility required the plasma membrane localization of AQP1 in the cancer cells. The block of migration by combined treatment with AqB011 and bacopaside II was significantly reduced following knockdown of AQP1 via siRNA transfection in HT29 cells. These data showed that the effectiveness of the blockers AqB011 and bacopaside II correlated with the levels of AQP1 expression in the membrane. AqB011 was not effective in blocking cell migration and invasion in SW480 as compared to HT29, an effect consistent with the low membrane levels of AQP1 measured in SW480. The fast rate of two dimensional migration in the low-AQP1-expressing SW480 cells suggested alternative mechanisms can drive fast movement, but interestingly the high surface velocity did not translate into an increased invasive potential across extracellular matrix. The higher invasive potential correlated with the high-AQP1-expressing HT29 cells. In both cell lines, combined block of the AQP1 ion channel and water pores was more potent in impairing motility across colon cancer types than single agents alone. The combined agents were able to restrain migration in the SW480 line, which escaped control by either single agent alone. The ability of SW480 cells to show normal wound closure with AqB011 or bacopaside II suggested these blockers did not act by causing non-specific disruption of cell membrane transport, metabolism, cytoskeletal structure, or other essential processes.

SW480 cells showed uniformly fast two-dimensional migration rates minimally affected by AqB011, suggesting that this class of cells achieves rapid migration via mechanisms largely independent of AQP1. The small amount of AQP1 that was seen in the SW480 plasma membrane might contribute to parallel roles such as steering, as needed to maintain a consistent direction of movement, or other functions. Low levels of AQP1 in the SW480 plasma membrane might contribute in small part to the enhanced cell motility. Contributions of other classes of AQPs remain to be explored; for example, SW480 cells express high levels of AQP5^60–62^. The role of the intracellular pool of AQP1 in SW480 cells remains to be defined. For example, in rat cholangiocytes, secretin stimulates translocation of AQP1 from vesicules to the plasma membrane and increases osmotic water permeability, an effect blocked by mercuric chloride^63^. Future work might identify signals that induce trafficking of intracellular AQP1 to the membrane, and alter motility in cancer lines.

Two-dimensional migration and three-dimensional invasiveness rely on different mechanisms. Surprisingly, bacopaside II alone strongly increased the invasiveness of both HT29 and SW480 cell lines, contradicting expectations based on wound closure assays. AqB011 fully reversed the pro-invasive effects of bacopaside II in HT29 cells, and partially reversed the enhanced invasion in SW480, in a pattern that was consistent with the levels of AQP1 membrane localization. Bacopaside II is a chemically complex molecule with a triterpene backbone^64^, unlikely to be selective for AQPs alone. Based on docking modelling^40^, the terpene is not involved in the AQP1 water pore block, which is mediated by the bacopaside sugar groups. Improved compounds in the future might use a trimmed version of the compound carrying the specific AQP1 channel blocking moeity. Terpenes increase the overall fluidity in phospholipid bilayers such as in parasites and mammalian cells^65–67^. Increased membrane fluidity correlates with augmented cancer invasiveness^68^, and invasive capacity of cancer cells can be suppressed by pharmacological reduction of membrane fluidity^69^. Unexpected effects of bacopaside on invasiveness reported here might be due to the membrane-fluidizing effects of the terpene component; this mechanism remains to be tested. However, bacopaside II appeared to reduce cell size and promote separation of individual cells from adjacent cells, suggesting a boost in invasiveness also might come from reduced cell-to-cell adhesion and decreased cell volumes, which could facilitate movement through narrow passages. The chemotactic gradient imposed by serum might restore the capacity for directional movement which appeared to be lost following bacopaside II treatment. At high doses bacopaside II is cytotoxic; at non-toxic doses it inhibits colon cancer growth by inducing cell cycle arrest, apoptosis^43^, and reducing angiogenesis^70^. Thus, bacopaside II and its metabolites *in vivo* are likely to affect a diverse array of processes, which could account for some of the observed effects on cell migration and invasion, as well as its beneficial effects as a traditional medicinal herb in a variety of applications^71,72^.

Results here are the first to show that AQP1 ion channel blocker AqB011 reduces colon cancer cell invasiveness *in vitro*, and to show that sensitivity to this agent depends on AQP1 localization in the plasma membrane. In summary, AQP1 water fluxes and ion conductance appear to exhibit a coordinated role in facilitating cell migration in AQP1-dependent cancer cell lines. Combined pharmacological block of both the AQP1 water and ion channels in HT29 and SW480 colon cancer cells amplified the inhibition of 2D cell migration, as compared with effects of either inhibitor alone. The prospect of a cooperative role between the AQP1 water flux and ion conductance is promising, in that lower doses of two compounds when combined could produce a beneficial level of cell migration impairment. Combined AQP1 inhibitors could act on target cells (such as migrating cancers) that require both the AQP1 water and ion channel activities, while minimizing side effects on other cells and tissues by being applied at lower concentrations. Future work is needed to explore effects of AQP1 inhibitors on other cancer cell types, to optimize bacopaside-related compounds for modulating AQP1 water flow, and to test the effectiveness of AQP1 agents in restraining metastasis *in vivo*.

## Methods

### Cell lines

Human colorectal adenocarcinoma cell lines HT29 (ATCC HTB-38TM) and SW480 (ATCC CCL-228TM) were cultured in T-75 plates in Dulbecco’s Modified Eagle Medium (DMEM; Life Technologies, Grand Island, NY, USA) supplemented with 10% fetal bovine serum (FBS; Life Technologies), 1% Gibco GlutaMAX (Life Technologies) and 100 units/ml each of penicillin and streptomycin (Life Technologies). Cell cultures were grown at 37 °C in a humidified 5% CO_2_ incubator.

### Quantitative PCR analysis of AQP1 expression

Cells plated in triplicate wells at 4 × 10^5^ cells/well were incubated at 37 °C in a humidified 5% CO_2_ incubator overnight. Total RNA was extracted using PureLink™ RNA Mini Kit (Invitrogen™); 1 µg total RNA was used for cDNA synthesis. cDNA was synthesized using QuantiTect® Reverse Transcription Kit (Qiagen®). cDNA was quantified using NanoDrop™ (Life Technologies); 50 ng cDNA was used in the polymerase chain reaction. Real-time qRT-PCR analyses were performed using SYBR™ Green PCR Master Mix (Applied Biosystems™) in a final volume of 10 µl with StepOne Plus™ Real-Time PCR system (Applied Biosystems™). Data were analysed by StepOne Plus™ Real-Time PCR software v2.3. The primer sequences for AQP1 were forward: 5′-CGCAGAGTGTGGGCCACATCA-3′, and reverse: 5′-CCCGAGTTCACACCATCAGCC-3′, amplifying a product of 217 bp. *RPS13* was used as a standard and target mRNA levels relative to *RPS13* were calculated using the formula 2^−ΔCT^ ^73^.

### Immunofluorescence

Cells were seeded 2–3 days prior to cell fixation on 8-well uncoated Ibidi® µ-Slides (Ibidi, Munich, Germany) at a density of 5 × 10^4^ cells/well. Once 80% confluent, cells were stained with MemBrite™ Fix Cell Surface Staining Kit (Biotium, Fremont, CA, USA; cat # 30093), and purchased from Gene Target Solutions (Dural, NSW, Australia). Membrane staining was performed prior to cell fixation, as per manufacturer’s instructions. Cells were fixed with 1:1 (v/v) acetone and methanol at −20 °C and incubated for 15 min at room temperature. Immunofluorescence staining was conducted as previously described by Wardill *et al*.^74^. The AQP1 primary antibody used for immunofluorescence was H-55 rabbit polyclonal (Santa Cruz Biotechnology, Dallas, TX, USA). The secondary antibody was Goat anti-Rabbit IgG (H + L) Cross-Adsorbed Secondary Antibody, Alexa Fluor 568 (Life Technologies; cat# A-11011). For nuclear staining, cells were incubated for 10 minutes at room temperature in a 1:1000 dilution of Hoechst 33258 (cat # 861405; Sigma-Aldrich, St. Louis, MO). The µ-Slide was imaged using an Olympus FV3000 Confocal Microscope. For the signal emitted by Hoechst 33258, excitation (Ex = 405 nm) and emission (Em = 461 nm) settings were used. For Alexa Fluor 568, settings were Ex = 561 nm and Em = 603 nm. For MemBrite™, settings were Ex = 488 nm and Em = 513 nm. Fiji (ImageJ) software (U.S. National Institutes of Health) was used to measure relative intensities of AQP1 signal standardized to membrane marker signal as a function of cross-sectional distance per cell as previously described^75^.

### AQP1 Inhibitors

The bumetanide derivative AqB011 (*Aq*uaporin ligand; *B*umetanide derivative; number *11* in a series) was synthesized by Dr Gary A. Flynn (SpaceFill Discovery LLC, West Yellowstone, MT, USA)^34^. Powdered AqB011 was dissolved in dimethyl sulfoxide (DMSO) to create 1000x stock solutions and diluted in culture medium to final concentrations for testing in the circular wound closure^76^, transwell invasion, live cell imaging, and alamarBlue assays. Bacopaside II was purchased from Sigma-Aldrich (St. Louis, MO), solubilized in methanol to yield 100x stock solutions, and stored at −20 °C in an airtight vial to minimize evaporation. For experimental use, bacopaside II stocks were diluted at 1/100 in culture medium. A combination of DMSO (1 μL/mL) and methanol (10 μL/ml) in culture medium was used as the vehicle control.

### Circular wound closure assay

Circular wound closure assays were performed using methods described by De Ieso and Pei^76,77^. In brief, cells were plated at 1 × 10^5^ cells/mL in DMEM culture medium with GlutaMAX and antibiotics (as above), reduced serum (2% FBS), and 400 nM of the mitotic inhibitor 5-fluoro-2′-deoxyuridine (FUDR). A confluent monolayer was achieved at 2–3 days following plating; circular wounds were created with a sterile p10 pipette tip. After washing two to three times with phosphate-buffered saline to remove cell debris, media were applied with and without AQP inhibitors or vehicle in low serum (2% FBS) DMEM with FUDR for the wound closure assay. Complete wounds were imaged at 10x magnification with a Canon 6D camera on a Nikon inverted microscope. Images were standardized using XnConvert software, and wound areas were quantified using Fiji software (ImageJ; version 1.51 h; U.S. National Institutes of Health). Closure was calculated as a percentage of the initial wound area for the same well as a function of time. All experiments were repeated in duplicate wells.

### Cytotoxicity assay

Cell viability was quantified using an alamarBlue assay^78^, following manufacturer’s guidelines (Life Technologies). Cells were plated at 10^5^ cells/mL in 96-well plates, in the same FUDR-containing low serum culture media as used in the migration assays. At 12–18 hours after plating, treatments were applied, and cells were incubated 24 hours. At 24 hours, cells were treated with 10% alamarBlue solution for 1–2 hours. Fluorescence signal levels were measured with a FLUOstar Optima microplate reader for control and treatment groups. Mercuric chloride (HgCl_2_) served as a positive control for inducing cytotoxic cell death, and a no-cell control was included to confirm low background fluorescence.

### Small interfering RNA transfection

The experiment was performed using a similar method as previously published^79^. Prior to transfection, HT29 cells were grown to 30% confluence in DMEM medium with 10% FBS. DharmaFECT 1 transfection reagent (T-2001-01; Dharmacon) was used to transfect Ambion Silencer Select AQP1 siRNA (siRNA1; 4390824; Life Technologies), Dharmacon ON-TARGETplus AQP1 siRNA (siRNA2; J-021494-05-0002; Dharmacon), and scrambled siRNA (4390843; Life Technologies). Each siRNA was administered at a concentration of 50 nM. Cells were incubated in 5% CO_2_ at 37 °C for 48 hours prior to wound closure assay and quantitative reverse-transcription polymerase chain reaction (RT-PCR) analyses.

### Live cell imaging

Cells were seeded on eight-well uncoated Ibidi μ-Slides (Ibidi) at a density of 1 × 10^5^ cells/mL. A confluent monolayer was achieved 2–3 days after plating. Cells were conditioned in low serum culture medium (2% FBS) in the presence of FUDR (400 nM) for 12–18 hours before wounding. Three circular wounds were created in each well using techniques described above for the wound closure assays. Slides were mounted on a Nikon Ti E Live Cell Microscope (Nikon, Tokyo, Japan) in an enclosed humidified chamber kept at 37 °C with 5% CO_2_. Images were taken at 30-minute intervals for 24 hours, using Nikon NIS-Elements software. AVI files were exported from NIS-Elements and converted into TIFF files using Fiji (ImageJ). Converted files were analyzed using Fiji software^80^ with the Manual Tracking plug-in. Total distance per cell was calculated as the cumulative distance travelled over the full duration of the experiment. Displacement was calculated as the net distance travelled between the first and last time points.

### Quantification of cell morphology

Images generated from the circular wound closure assay at the 24 hour time point were processed using FIJI. Images were first converted into binary file Process > Binary > Make Binary) and then skeletonized (Process > Binary > Skeletonize). Individual cells were randomly chosen for analysis from the leading edge of the wound. The total skeleton length of an individual cell was measured (Analyze > Measure) and classified as cell “complexity”.

### Transwell invasion assay

Assays were performed using 6.5 mm Corning^®^ Transwell^®^ polycarbonate membrane cell culture inserts with 8 μm pore size (cat #3422; Sigma-Aldrich, St. Louis, MO), as previously described^81^. The upper surface of the filter was coated with 40 μL of water-diluted extracellular matrix (ECM) gel from Engelbreth-Holm-Swarm murine sarcoma (final concentration 25 μg/mL; Sigma-Aldrich, St. Louis, MO), and left to dehydrate overnight, and rehydrated 2 hours prior to cell seeding with 50 μL of serum-free DMEM per transwell insert. Cells were grown to approximately 40% confluence under normal conditions, and transferred into reduced serum (2% FBS) medium for 32–34 hours prior to seeding. Cells were harvested, resuspended in serum-free DMEM, and 2.5 × 10^5^ cells in 100 μL was added to the upper chamber (total 150 μL of cell suspension per transwell, including 50 μL of rehydration medium added earlier). To the lower chamber, 600 μL of pharmacological treatment in DMEM supplemented with 10% serum (chemoattractant) was added, and cells were incubated for 24 hours at 37 °C in 5% CO_2_. Non-migrated cells were scraped from the upper surface of the membrane with a cotton swab; migrated cells remaining on the bottom surface were counted after staining with crystal violet^82^.

### Statistical analyses

Statistical analyses performed with GraphPad Prism 7.02 software involved one-way ANOVA and post-hoc Bonferroni tests. Statistically significant outcomes are represented as (*)p < 0.05, (**)p < 0.01, (***)p < 0.001, or (****)p < 0.0001; NS is not significant; other characters indicating significance (# and +) use the same pattern for defining p values. All data are presented as mean ± standard error of the mean (SEM); *n* values for independent samples are indicated in italics above the x-axes in histogram figures.

## Supplementary information

HT29 video

SW480 video

## Data Availability

All data generated or analysed during this study are included in this published article (and its Supplementary Information files).
